# Use of a Fission Yeast Platform to Identify and Characterize Small Molecule PDE Inhibitors

**DOI:** 10.3389/fphar.2021.833156

**Published:** 2022-01-17

**Authors:** Charles S. Hoffman

**Affiliations:** Biology Department, Boston College, Chestnut Hill, MA, United States

**Keywords:** phosphodiesterase, selective PDE inhibitors, inflammation, *Schizosaccharomyces pombe*, high throughput screen (HTS), *Caenorhabditis elegans*, parasitic nematodes

## Abstract

Cyclic nucleotide phosphodiesterases (PDEs) have been proven to be targets for which highly selective and potent drugs can be developed. Mammalian genomes possess 21 genes whose products are pharmacologically grouped into 11 families; however related genes from pathogenic organisms display sufficient divergence from the mammalian homologs such that PDE inhibitors to these enzymes could be used to treat parasitic infections without acting on the related human PDEs. We have developed a platform for expressing cloned PDEs in the fission yeast *Schizosaccharomyces pombe*, allowing for inexpensive, but robust screening for small molecule inhibitors that are cell permeable. Such compounds typically display the expected biological activity when tested in cell culture, including anti-inflammatory properties for PDE4 and PDE7 inhibitors. The genetic pliability of *S. pombe* also allows for molecular genetic screens to identify mutations in target PDE genes that confer some resistance to these inhibitors as a way of investigating the PDE-inhibitor interaction. This screening method is readily accessible to academic laboratories as it does not require the purification of large quantities of a target protein. This allows for the discovery and profiling of PDE inhibitors to treat inflammation or of inhibitors of targets such as pathogen PDEs for which there may not be a sufficient financial motivation for pharmaceutical companies to identify selective PDE inhibitors using more traditional *in vitro* enzyme-based screening methods.

## Introduction

Cyclic nucleotide signaling has been associated with a wide range of biological processes including oncogenesis, cognition, steroidogenesis, and inflammation, to name but a few (Lerner and Epstein, 2006; Conti and Beavo, 2007). The signaling molecules 3’-5’ cyclic adenosine monophosphate (cAMP) and 3’-5’ cyclic guanosine monophosphate (cGMP) are produced by adenylyl and guanylyl cyclases, respectively, in response to external signals, and are hydrolyzed to 5’AMP and 5’GMP by cyclic nucleotide phosphodiesterases (PDEs). Mammalian genomes possess 21 Class I PDE genes that are pharmacologically grouped into 11 families based on their specificity for cAMP and/or cGMP, structural features outside of the catalytic domains, and response to small molecules and other regulatory mechanisms. With regard to inflammation, therapeutic benefits have been demonstrated or proposed for inhibitors of PDE1, PDE2, PDE3, PDE4, PDE5, PDE7, PDE8, and PDE11 (Bender and Beavo, 2006; Horvath et al., 2006; Vignozzi et al., 2013; Maurice et al., 2014; Pathak et al., 2017). This article is not intended to replace or repeat the many excellent reviews on these PDEs and their inhibitors. It is instead focused on two themes: 1) That there is a place for academic laboratories to make contributions to the discovery and development of PDE inhibitors using methods not traditionally employed by the pharmaceutical industry that has dominated this field, and 2) that the PDEs of parasitic organisms can serve as drug targets to treat infections that involve a host’s immune system and produce an inflammatory response (thus, an appropriate topic for this volume).

High throughput screens (HTS) as part of drug development had long been the domain of large pharmaceutical companies, largely due to a lack of funding for such academic research, and only entered the academic sphere in the late 1990s. Relatively few institutions had the wherewithal to create the infrastructure needed and to recruit the experienced staff to support academic research that utilized HTS as a method to identify compounds able to affect the activity of target proteins or pathways. In addition, there was little extramural support for such work. In 2002, the National Cancer Institute established the Initiative for Chemical Genetics at the Broad Institute to support academic HTS with a particular focus on cancer therapies (Tolliday et al., 2006). In 2004, the NIH established its own NIH Chemical Genetics Center (NCGC), which became the National Center for Advancing Translational Sciences (NCATS) in 2008. Along with the NCGC, NIH supported the development of nine additional screening centers around the United States and created the Molecular Libraries Screening Center Network (MLSCN) to support access to these facilities by academic laboratories that would be the end users in need of a compound to target a specific protein or pathway of interest to test a research hypothesis (Huryn and Cosford, 2007). Shortly after that, the NIH initiated the Molecular Libraries Probe Production Centers Network (MLPCN) to first encourage academic laboratories to create and contribute novel compounds to a central library and then to support screening of this library by academic end users through these various centers. This has led to many successful campaigns to discover new chemical probes for a wide range of targets (Hasson and Inglese, 2013). However, grant support for these screens generally paid for costs incurred by the screening centers or for the development of a robust screening assay for a specific target as part of a hypothesis-driven project. It was not intended for laboratories trying to develop novel screening methods to identify compounds for which they would not be the end user, and thus failed to fully capitalize on the possible diversity of screening methods that could be developed within academia.

It is not unusual for model organism geneticists to run up against the ethos behind the hypothesis-driven science mandate. However, as Fred Winston and Doug Koshland note, many fundamental breakthroughs in science came about from unbiased mutant hunts for which the researchers had no idea in advance where the genetics would take them (Winston and Koshland, 2016). Fred Winston expressed this idea in a more light-hearted manner at a 2000 “Yeast Fest” Symposium honoring the 60^th^ birthday of his postdoctoral advisor, the yeast pioneer Gerald Fink. “Every project in the Fink lab was based on the same hypothesis: Maybe we can find some interesting mutants.” These projects launched the careers of several members of the National Academy of Sciences and led to fundamental discoveries in mechanisms of transcription, translational regulation, retrotransposition, fungal pathogenesis, signal transduction, and cell biology (Roeder and Fink, 1980; Hinnebusch and Fink, 1983; Winston et al., 1984; Boeke et al., 1985; Rose and Fink, 1987; Elion et al., 1990; Liu et al., 1994; Bartel and Fink, 1995; Madhani and Fink, 1998; Verstrepen et al., 2005).

This chapter describes my laboratory’s development of a high throughput screen for PDE inhibitors targeting mammalian PDEs that are heterologously expressed in the fission yeast *Schizosaccharomyces pombe* (Hoffman et al., 2015). Due to the required characteristics of the compounds identified in this manner, described below, we too had a single hypothesis for all of our screens: “Maybe we can find some interesting compounds that others can use in their research without the need for further medicinal chemistry”.

## Features of Yeast-Based High Throughput Screens

There are a variety of screening methods employed in HTS, each with its strengths and weaknesses. For example, screens utilizing *in vitro* enzyme assays can readily identify compounds that act on the target protein, however there may be little information initially to guide prioritization (Inglese, 2002). Most of the hit compounds will also show no activity in cell culture or on the target organism due to a lack of cell permeability and will require significant modifications to facilitate cell entry. In addition, *in vitro* enzyme assays tend to be relatively expensive due to the need for a purified protein target and an assay that requires a suitable substrate to provide a readout of protein activity (Denny and Steel, 2015; Denny, 2018). In comparison, a phenotypic screen using a target cell or organism will detect cell-permeable compounds and is less likely to require expensive reagents, however it may be moderately expensive or difficult to culture the target cells/organism and the identity of the protein affected by a hit compound may be hard to determine (Denny and Steel, 2015; Denny, 2018). Meanwhile, screens that involve yeast strains that have been engineered to express a target protein from another organism possess the strengths of both biochemical assays and phenotypic assays. Similar to phenotypic assays, only cell-permeable compounds will be identified as hit compounds and the assay is unlikely to require expensive reagents. In addition, culturing yeast is an inexpensive endeavor and there are a variety of growth media and conditions available that may be intrinsic to the detection method. However, by screening for compounds that act on a foreign protein expressed in the host strain, there is an added feature of target identification that is the strength of the *in vitro* enzyme assay method. This involves assessing candidate hit compounds with a counter-screening strain, i.e., one that lacks the original target protein, possibly by expressing the endogenous yeast homolog, to screen out two types of artifactual hits. The first type is compounds that act on a host protein that would mimic the effect of acting on the target foreign protein (Denny and Steel, 2015; Denny, 2018). The second type is compounds that act via reporter interference, creating a specific artifact in the reporter readout (Hasson and Inglese, 2013). In both cases, the authentic hit compounds should not show activity when tested on the counter-screening strain, while artifactual hit compounds will appear to be active on this strain as well.

## Yeast-Based Phenotypes Associated With cAMP Signaling

Both the budding yeast *Saccharomyces cerevisiae* and the fission yeast *S. pombe* utilize cAMP signaling to control important aspects of cell growth and stationary phase survival. However, one key difference between these yeasts is the fact that cAMP is essential for *S. cerevisiae*, but not *S. pombe*, as *S. cerevisiae* mutants in the adenylyl cyclase gene *CYR1* arrest in G1 of the cell cycle, while similar *S. pombe* mutants do not (Matsumoto et al., 1983; Maeda et al., 1990). Meanwhile, mutations in both of the *S. cerevisiae* PDEs genes, *PDE1* and *PDE2*, lead to both a reduction of viability and a severe loss of heat shock resistance in stationary phase cells (Nikawa et al., 1987). Similarly, a mutation in the sole *S. pombe* PDE gene, *cgs2*, leads to a rapid loss of viability in stationary phase cells and defects in both mating and in glucose-starvation-induced expression of the *fbp1* gene that encodes the gluconeogenic enzyme fructose-1-6-bisphosphatase (DeVoti et al., 1991; Hoffman and Winston, 1991). These phenotypes allow for detection of PDE activity from heterologously-expressed PDE genes, although the sensitivity to PDE inhibitors is significantly different in the two yeasts due to differences in the phenotypes used in such studies.

### Saccharomyces cerevisiae

The ability of PDEs to lower cAMP levels in *S. cerevisiae* was used to clone mammalian PDE genes and to isolate mutations in a *PDE4* gene that confer resistance to the PDE4 inhibitor Rolipram. These studies exploited the fact that while actively growing *S. cerevisiae* cells are sensitive to a severe heat shock, stationary phase cells are resistant (Walton and Pringle, 1979). As mentioned above, deletion of both the *S. cerevisiae PDE1* and *PDE2* genes results in cells that fail to acquire heat shock resistance upon growth to saturation, which would normally lead cells to enter stationary phase. Screens of mammalian cDNA libraries that allowed expression of the cloned genes identified human PDE4A and PDE7A, as well as rat PDE4B, as clones that could restore heat shock resistance to strains lacking *PDE1* and *PDE2* or expressing a mutationally activated *RAS2* ^*val19*^, which hyperactivates the *S. cerevisiae* adenylyl cyclase, thus phenocopying the loss of PDE activity (Colicelli et al., 1989; Colicelli et al., 1991; Michaeli et al., 1993). Subsequent work with the rat PDE4B clone showed that the PDE activity in budding yeast could be inhibited by the PDE4 inhibitor Rolipram to restore heat shock sensitivity, although the concentration of Rolipram used was 500 μM (Pillai et al., 1994). This allowed for the isolation of plasmids expressing a mutant *PDE4B* allele that produced a Rolipram-resistant PDE as stationary phase cells expressing the mutant protein remained heat shock resistant in the presence of Rolipram. While these studies demonstrate the utility of a *S. cerevisiae* system to clone and study mammalian PDEs, the use of a stationary phase phenotype made this untenable for HTS for two reasons. First, a screen based on heat shock resistance cannot be readily scaled up for screening thousands of compounds due to the manipulations needed to assess the response to heat shock. Secondly, extremely high concentrations of compound are required to confer heat shock resistance due to the fact that stationary phase cells are less permeable than actively growing cells as was shown for sensitivity to hydrogen peroxide (Sousa-Lopes et al., 2004).

### Schizosaccharomyces pombe

Interest in *S. pombe* cAMP signaling came from two distinct factions. First, there were those whose primary interest was in the *S. pombe* Ras1 protein as a model for understanding Ras proteins and their roles in oncogenesis. It had been shown that *S. cerevisiae* adenylyl cyclase was regulated by Ras proteins (Toda et al., 1985), thus there was an interest in seeing if *S. po*mbe adenylyl cyclase was similarly regulated and possibly more amenable to studying Ras function. Surprisingly, it turned out that while Ras proteins act in the G-protein-mediated cAMP pathway in *S. cerevisiae*, Ras1 of *S. pombe* acts in the G-protein-mediated pheromone signaling pathway (Fukui et al., 1986). A detailed description of similarities and differences between the G-protein mediated glucose- and pheromone-signaling pathways in *S. cerevisiae* and *S. pombe* can be found elsewhere (Hoffman, 2005). The second foray into cAMP signaling came from two labs using genetic approaches to study two completely different biological processes without prior knowledge of a role for cAMP signaling. It should be mentioned here that PKA is the only *S. pombe* protein known to be controlled by cAMP and thus all cAMP-regulated processes are controlled by PKA. In the first, mutations in the *cgs1* and *cgs2* genes, encoding the PKA regulatory subunit and the sole *S. pombe* PDE protein, were identified by their ability to suppress meiotic catastrophe that occurs in haploid *pat1*/*ran1* mutant cells as they attempt to enter meiosis with just a single set of chromosomes (DeVoti et al., 1991). From this study, it was shown that high PKA activity inhibits mating and leads to a rapid loss of viability as cells grow to saturation due to a failure to enter stationary phase. Meanwhile, our interest in the mechanism by which high levels of glucose in the growth medium repress transcription of the *fbp1* gene led to a genetic screen for mutants that continue to transcribe an *fbp1-ura4* reporter while growing in a glucose-rich medium (Hoffman and Winston, 1990). The first of these genes to be cloned was *git2*, which encodes adenylyl cyclase (Hoffman and Winston, 1991). Of the remaining nine *git* genes, six encode proteins required for adenylyl cyclase activation, while a seventh gene encodes the catalytic subunit of PKA (Nocero et al., 1994; Jin et al., 1995; Landry et al., 2000; Welton and Hoffman, 2000; Schadick et al., 2002; Kao et al., 2006; Alaamery and Hoffman, 2008). These genes include *git8*/*gpa2* encoding the G*α* subunit of a heterotrimeric G protein and *git5* encoding the G*β* subunit of this G protein. Using the Git5 G*β* as a bait in a two-hybrid screen, the *git11* gene encoding the Gγ subunit was later identified (Landry and Hoffman, 2001).

Studies of the role of cAMP signaling and PKA activity in *S. pombe* have revealed a variety of phenotypes that allow one to distinguish between cells that have high or low PKA activity (Table 1). Some of these phenotypes are based on the construction of reporter genes fused to the *fbp1* promoter and are therefore transcriptionally repressed by PKA activity. The *E. coli lacZ* gene encodes *β*-galactosidase, which can be readily measured using a colorimetric assay on protein extracts (Hoffman and Winston, 1990). The *Aequorea victoria* green fluorescent protein (GFP) gene encodes a protein that can be assessed by fluorescence spectroscopy, fluorescence microscopy, or fluorescence scanning using live cells (de Medeiros et al., 2013; Getz et al., 2019). *The S. pombe ura4* gene encodes OMP decarboxylase, an enzyme acting in the uracil biosynthetic pathway. This is a unique reporter in that it acts as a selectable marker in medium lacking uracil (as expression is required for growth) and a counterselectable marker in medium containing the pyrimidine analog 5-fluoroorotic acid (5FOA; as expression prevents growth) (Boeke et al., 1984). Thus, PKA repression due to elevated cAMP levels results in cells that are 5FOA-resistant (5FOA^R^), but require uracil as a supplement to the growth medium (Ura^−^). Meanwhile, cells that have reduced cAMP levels are sensitive to 5FOA (5FOA^S^), but are able to produce enough uracil to support growth in medium lacking uracil (Ura^+^). Other phenotypes associated with high or low PKA activity are intrinsic to *S. pombe* and are not based on the presence of a reporter construct. These include the length of cells at the time of forming a fission plate, which immediately precedes cytokinesis, the ability to grow in the presence of 1M KCl, mating competence, and the ability to successfully enter stationary phase (Table 1). This wealth of phenotypes, together with the fact that PKA activity is not essential for *S. pombe* cell viability, has allowed us to construct a large collection of strains that express mammalian PDEs, adenylyl cyclases, and the human GNAS G*α*_S_ that stimulates the nine mammalian transmembrane adenylyl cyclases (Ivey et al., 2008; Alaamery et al., 2010; Demirbas et al., 2011a; Demirbas et al., 2011b; Ceyhan et al., 2012; Demirbas et al., 2013; de Medeiros and Hoffman, 2015; Getz et al., 2019).

**TABLE 1:** *S. pombe* phenotypes associated with PKA activity.

Process or reporter	High PKA phenotype	Low PKA phenotype
Doubling time	∼3 h	4–6 h
Cell length at septation	15–17 microns	10–12 microns
Sexual development	Partial sterility	Sexually competent
Stationary phase entry	Defective: rapid cell death	Successful entry
Salt stress sensitivity	KCl-resistance	KCl-sensitivity
*fbp1-lacZ*	Low *β*-gal activity	High *β*-gal activity
*fbp1-GFP*	Low fluorescence	High fluorescence
*fbp1-ura4*	5FOA^R^; Ura^−^	5FOA^S^; Ura^+^

Phenotypes that vary in S. pombe as a function of PKA activity.

## Fission Yeast-Based PDE Inhibitor Screens

### Critical Features of the fbp1-ura4 Based Screen

As seen in Table 1, PKA activity confers a positive growth phenotype in cells possessing the *fbp1-ura4* reporter when growing in medium containing 5FOA. Thus, if a strain expresses a PDE that lowers cAMP or cGMP levels to prevent 5FOA^R^ growth, inhibition of that PDE could convert it to a high PKA (5FOA^R^) phenotype. The 5FOA growth readout for PDE inhibitors requires hit compounds to possess many characteristics that are not needed for a compound to show activity in an *in vitro* enzyme assay-based screen. While this means that some compounds capable of inhibiting the target PDE will be missed, it can be seen as a positive feature as a screen that only relies on PDE binding and inhibition in a biochemical reaction is likely to produce an overwhelming number of candidate molecules that will require additional evaluation to prioritize those best suited for further development (Inglese, 2002). Specifically, hit compounds in this yeast-based growth assay must be cell permeable, moderately soluble in the aqueous 5FOA medium, and remain chemically stable during the 48 h growth period. In addition, since the readout is enhanced growth, these compounds must not inhibit any of the roughly 1,500 proteins that are essential for growth, demonstrating a degree of selectivity for the target PDE that would bode well for drug development. Table 2 provides a summary of the screens we have carried out using strains that express PDE4, PDE5, PDE7, PDE8, or PDE11 proteins, along with the screening conditions and the compounds highlighted from each screen.

**TABLE 2:** Summary of HTS using fission yeast 5FOA growth assays.

PDE family	Target gene(s)	Number of compounds screened	Key features of screen (all strains lack *S. pombe* Cgs2 PDE)	Identified compound(s)	Citation
PDE4	Murine PDE4A1 Murine PDE4B3	3,120	Hosts lack Gpa2 G*α* that activates adenylyl cyclase	Rolipram Zardaverine Imperatorin	Ivey et al. (2008)
PDE4	Murine PDE4A1 Murine PDE4B3 Rat PDE4A5	74,090 78,895 39,923	Hosts lack Gpa2 G*α* that activates adenylyl cyclase	BC54	de Medeiros et al. (2017)
PDE7	Human PDE7A1	48,176	Host lacks Gpa2 G*α* that activates adenylyl cyclase	Canrenone Androsterone acetate BC11 BC30 BC39	Alaamery et al. (2010)
PDE7	Human PDE7A1	48,176	Host lacks Gpa2 G*α* that activates adenylyl cyclase	BC12	Xu et al. (2016)
PDE7	Human PDE7A1	48,176	Host lacks Gpa2 G*α* that activates adenylyl cyclase	BC54	de Medeiros et al. (2017)
PDE5	Bovine PDE5A1	60 hit compounds from PDE4 and PDE7 screens	Host lacks adenylyl cyclase activity; 5FOA medium contains 200 μM cGMP	BC76	Demirbas et al. (2011b)
PDE8	Human PDE8A1	222,711	Host lacks adenylyl cyclase activity; 5FOA medium contains 40 μM cAMP	BC8-15	Demirbas et al. (2013)
PDE11	Human PDE11A4	198,382	Host lacks adenylyl cyclase activity; 5FOA medium contains 40 μM cGMP	BC11-15 BC11-19 BC11-28 BC11-38	Ceyhan et al. (2012)

### PDE4 Inhibitors

The Rolipram-sensitive PDE4 enzymes, encoded by the *PDE4A*, *PDE4B*, *PDE4C*, and *PDE4D* genes, have long been associated with inflammation and immune cell function (Bender and Beavo, 2006; Lerner and Epstein, 2006; Conti and Beavo, 2007; Maurice et al., 2014). As a cAMP-specific PDE family, we focused on this family during the development phase of the fission yeast-based system, since it was not yet known that cGMP-hydrolyzing PDEs could also be studied in this manner (Ivey et al., 2008). Our pilot study identified the balance between cAMP production and PDE activity needed to generate strains that would be sensitive to PDE inhibitors. Strains lacking both the *S. pombe gpa2* G*α* that stimulates adenylyl cyclase and the *cgs2* PDE gene are 5FOA^R^, but become 5FOA^S^ upon expression of either murine PDE4A1 or PDE4B3 (Ivey et al., 2008). This allowed for the detection of PDE4 inhibitors that confer 5FOA^R^ growth from a collection of 3,120 known bioactive compounds from the Prestwick and Microsource Spectrum libraries in 50 μL cultures exposed to ∼20 μM compound (Table 2). Hit compounds included Rolipram and the dual selective PDE3/4 inhibitor Zardaverine. Also identified in this screen was the furanocoumarin Imperatorin, which was verified as a PDE4 inhibitor by measuring its impact on cAMP levels in the *S. pombe* screening strains. As both Rolipram and Imperatorin had been independently shown to inhibit HIV replication, this study provided a likely mechanism of action for Imperatorin (Angel et al., 1995; Sancho et al., 2004). This study also showed that *S. pombe* strains could be screened in a 384 well format for PDE inhibitors, which was not possible using the *S. cerevisiae* system for studying PDE activity. The key differences between the two yeast systems are that 1) the *S. pombe* phenotype based on *fbp1-ura4* expression allowed for the study of actively growing cells that require no additional manipulation to detect a phenotypic change, unlike the need to carry out a heat-shock experiment on patches of *S. cerevisiae* cells and 2) actively growing *S. pombe* cells in liquid culture are likely more permeable to compounds than are *S. cerevisiae* cells grown as a dense patch on a plate.

### PDE7

Similar to PDE4 enzymes, PDE7 enzymes, encoded by the *PDE7A* and *PDE7B* genes, are cAMP-specific and are involved in immune cell function (Bender and Beavo, 2006; Lerner and Epstein, 2006; Conti and Beavo, 2007; Maurice et al., 2014). Expression of either PDE7A or PDE7B in *S. pombe* confers sufficient PDE activity to construct HTS strains based on endogenous cAMP production from the *S. pombe* adenylyl cyclase (Alaamery et al., 2010). A screen of 48,176 compounds led to the identification of 61 compounds that produced an absorbance of greater than half that of the positive control wells in which 5 mM cAMP was added to the 5FOA medium to activate PKA. These included steroids canrenone and androsterone acetate, as well as structurally-related podocarpanes that possess a series of three fused six-carbon rings that resemble a substructure of steroids (Table 2). One of the more unusual compounds with regard to chemical structure, designated BC30, showed a slight preference for PDE7A over PDE7B both in the 5FOA growth assay and in subsequent *in vitro* enzyme assays (Alaamery et al., 2010). Surprisingly, a control compound, BRL50481 showed a substantial preference for PDE7A relative to PDE7B in both the 5FOA growth assays and in *in vitro* enzyme assays. BRL50481 has been described as a PDE7 inhibitor, although the only data presented in the original paper was generated using PDE7A1, and it was suggested that BRL50481 would likely have similar activity against PDE7B (Smith et al., 2004). Meanwhile, our *in vitro* assays reveal an 80-fold preference for PDE7A over PDE7B. When tested for anti-inflammatory activity on activated pro-monocytic U937 cells, BC30 substantially reduced TNF*α* release and significantly enhanced the anti-inflammatory effect of Rolipram treatment (all compounds tested at 10 μM). Meanwhile BRL50481 displayed little activity on its own and showed only modest enhancement of the effect of Rolipram (Alaamery et al., 2010). Based on these data, BRL50481 is a PDE7A inhibitor rather than a PDE7 inhibitor.

Another strength of this platform is the ease with which chemical analogs of hit compounds can be screened to identify compounds with enhanced or reduced PDE inhibitory activity. While the value of finding more potent PDE inhibitors is obvious, we believe that the identification of inactive analogs can be equally important. For example, the PDE7 inhibitor, BC12 (Table 2), displays a remarkable ability to fully inhibit IL-2 production by stimulated Jurkat T cells, well beyond anything we found in the literature for PDE inhibitors (Xu et al., 2016). Therefore, we were not surprised to see that a derivative, BC12-4, which lacks the ability to inhibit PDE7, is also able to completely block an IL-2 response in Jurkat cells. Such a control compound is critical to establishing that the biologically-relevant response to these compounds is actually due to PDE inhibition.

### PDE4/7

While PDE4 inhibitors have been long sought to treat a variety of conditions, it is only recently that three FDA-approved PDE4-targeting drugs have been developed that avoid the significant emetic response plaguing most PDE4 inhibitors (Phillips, 2020). It has been proposed that since both PDE4 and PDE7 enzymes play roles in inflammation, a dual specificity PDE4/7 inhibitor might be more readily tolerated at a therapeutic dose (Castro et al., 2005; Giembycz, 2005). From HTSs conducted with murine PDE4A1 and PDE4B3, rat PDE4A5 and human PDE7A1, we identified a potent PDE4/7 inhibitor, BC54 (Table 2) (de Medeiros et al., 2017). BC54 is equally effective against PDE7A and PDE7B as judged by both 5FOA growth assays and *in vitro* enzyme assays. Meanwhile, it outperforms the PDE4 inhibitor Rolipram and the PDE7A inhibitor BRL50481, both individually and in combination in anti-inflammatory assays of TNF*α* production by stimulated U937 cells and IL-2 production by stimulated Jurkat cells (de Medeiros et al., 2017). BC54 also outperforms a combination of Rolipram and BRL50481 in its ability to induce apoptosis in chronic lymphocytic leukemia cells from three patients (de Medeiros et al., 2017), supporting the promise of therapeutic benefit by a dual specificity PDE4/7 inhibitor. This study also featured a classic philosophy of yeast genetic studies, isolating suppressor mutations to further our understanding of a process (Prelich, 1999). In this case, a plasmid-based screen was carried out to identify mutations in the human *PDE4B2* open reading frame (ORF) that would confer some degree of resistance to BC54 as seen by the retention of the low PKA phenotype of colony formation on medium lacking uracil in the presence of BC54 (Table 1). Remarkably, one of the two mutations identified in this manner is equivalent to that of a mutation found using a plasmid-based screen of a cloned rat *PDE4B* ORF that confers resistance to Rolipram when expressed in *S. cerevisiae* (Pillai et al., 1994). Thus, the *S. pombe fbp1-ura4* reporter can be exploited in both chemical genetic screens to identify PDE inhibitors and in molecular genetic screens to find mutant alleles of target genes that produce compound-resistant PDEs.

### PDE4/PDE8

The PDE8 family, encoded by the PDE8A and PDE8B genes, is the third cAMP-specific family of PDE enzymes and plays roles in steroidogenesis and immune cell function (Bender and Beavo, 2006; Dong et al., 2006; Lerner and Epstein, 2006; Conti and Beavo, 2007; Shimizu-Albergine et al., 2012; Maurice et al., 2014). Expression of PDE8A from the *S. pombe* PDE locus produced significantly less PDE activity than did the PDE4 or PDE7 enzymes, such that a screening strain lacking the *S. pombe* adenylyl cyclase was used and PKA activation was achieved by adding 40 μM cAMP to the 5FOA medium, an amount that confers 5FOA^R^ growth to a strain devoid of PDE activity, but not to the strain expressing PDE8A (Demirbas et al., 2013). A screen of more than 220,000 compounds identified 11 compounds that could inhibit PDE8A and PDE8B with modest potency (<10 μM IC_50_). The most effective compound is a dual specificity PDE4/8 inhibitor, BC8-15 (Table 2, IC_50_ of 280 nM against PDE8A and 220 nM against PDE4A), although it also displays low micromolar activity against other PDEs. Consistent with a role for PDE8 in steroidogenesis, BC8-15 elevates testosterone production by primary Leydig cells from a wild type mouse (is there really such a thing?), but not from a mouse lacking both PDE8A and PDE8B (Demirbas et al., 2013). Finally, while BC8-15 inhibits several other PDEs at single digit micromolar concentrations, a comparison of BC8-15 with two derivatives that display unique *in vitro* PDE assay profiles when tested against PDEs from every family other than PDE6 shows that the ability to elevate progesterone release by MA-10 Leydig tumor cells correlates specifically with PDE8 inhibition (Demirbas et al., 2013). This result again demonstrates the value of profiling derivatives of hit compounds to characterize compounds that are less active than the original library compound.

### cGMP-Hydrolyzing Phosphodiesterases

Our initial emphasis was on cAMP-specific PDEs, as we were exploiting the *S. pombe* cAMP signaling pathway for this work. However, we stumbled across the fact that some PKA regulatory subunits bind cGMP nearly as well as they bind cAMP (Cytrynska et al., 1999; Frajnt et al., 2003). This is true of the *S. pombe* Cgs1 PKA regulatory subunit, allowing us to work with PDEs that are either more effective at hydrolyzing cGMP than cAMP or are uniquely active on cGMP. By using a screening strain that lacks the *S. pombe* adenylyl cyclase gene, we can control PKA through the addition of cGMP to the 5FOA growth medium (Demirbas et al., 2011b). This discovery led us to expand our strain collection to include strains that express mammalian PDEs from 10 of the 11 PDE families (with the exception of PDE6). Although we have not conducted HTSs using PDE5- or PDE9-expressing strains, which together with PDE6 represent the cGMP-specific PDEs, we have identified compounds that are effective PDE5 inhibitors among 60 hit compounds from screens for both PDE4 and PDE7 inhibitors (Table 2) (Demirbas et al., 2011b; Ceyhan et al., 2012). One such compound, BC76, displays an IC_50_ of 232 nM against PDE5 in an *in vitro* enzyme assay, while showing activity against PDE1, PDE4, PDE5, PDE8, PDE10, and PDE11 in 5FOA growth assays (Demirbas et al., 2011b). Ironically, *S. pombe* cells expressing PDE5, the target of erectile dysfunction drugs, and grown in the presence of cGMP dramatically elongate upon exposure to BC76 (Demirbas et al., 2011b), as PKA activity regulates the length of cells required to trigger mitosis (Table 1) (Navarro and Nurse, 2012; Mudge et al., 2014). No wonder *S. pombe* is sometimes referred to as a unicellular micromammal (Vyas et al., 2021)!

### PDE11

PDE11 is encoded by a single gene, *PDE11A*, and is a dual-specificity enzyme involved in regulating the release of the anti-inflammatory hormone cortisol in humans by the adrenal gland, as well as in neuroinflammation (Horvath et al., 2006; Pathak et al., 2017). While the PDE5 inhibitor tadalafil displays potent off-target activity against PDE11 (Weeks et al., 2005), it was not until we conducted a nearly 200,000 compound screen that four selective PDE11 inhibitors were identified displaying IC_50_ values ranging from 110 to 330 nM (Table 2) (Ceyhan et al., 2012). As the original clone of human PDE11A4 was expressed from the relatively weak *S. pombe* PDE (*cgs2*) promoter, the PDE activity was insufficient to allow for a HTS using endogenous cAMP production, thus the 5FOA assay was carried out using exogenous cGMP (40 μM) in the 5FOA growth medium. We have since expressed PDE11A4 from the more active *adh1* promoter (Russell and Hall, 1983). The increase in PDE activity allows us to conduct 5FOA assays in a strain that produces cAMP from a heterologously-expressed mammalian adenylyl cyclase (Getz et al., 2019), thus avoiding the need for exogenous cyclic nucleotides in the 5FOA medium. One compound from the HTS, BC11-38, elevates cAMP levels, PKA activity and cortisol release by H295R adrenocortical cells. In addition, four derivatives were characterized by *in vitro* enzyme assays, as well as in the H295R cell culture assays. Efficacy in H295R cell culture assays correlates with the IC_50_ values for PDE11 inhibition. Furthermore, these compounds fail to elevate cAMP levels and PKA activity in HeLa cells, which show little to no PDE11 transcript. Taken together, these results suggest that BC11-38 acts on H295R cells *via* PDE11 inhibition. Similar to the PDE8 study described above (Demirbas et al., 2013), this work takes advantage of derivatives of the initial hit compound to assess whether the biological activity is a function of the compounds’ ability to inhibit the target PDE and to rule out an off-target mechanism as was seen with the PDE7 inhibitor BC12 (Xu et al., 2016). Finally, medicinal chemistry is currently being performed using starting material from this screen in an effort to develop more drug-like PDE11 inhibitors. This is the only follow-on project to develop therapeutics based on the PDE inhibitors identified in these screens that I am aware of.

## Targeting Parasitic Nematode PDES

As this volume is devoted to immune regulation and inflammation, a discussion of PDEs from parasitic organisms may seem somewhat off topic, however the immune system and inflammation are intrinsically involved in infectious disease. Previously, we expressed in *S. pombe* PDE4, PDE8, and PDE11 family members from the blood fluke *Schistosoma mansoni*, whose genome includes members of all 11 Class I PDE families (Vyas et al., 2021). 5FOA growth assays were used to determine the relative ability of these enzymes to hydrolyze cAMP and cGMP as shown by an increase in amount of exogenous cNMP required to confer 5FOA^R^ growth (Munday et al., 2020). However, an even more fertile area of research could involve the treatment of infections by parasitic nematodes due to the existence of the well-established model organism nematode *Caenorhabditis elegans* (Corsi et al., 2015), whose genome includes only six Class I PDE genes. Thus, the study of these PDEs in *C. elegans* can provide insight into the efficacy of developing drugs to treat infections by roundworms, hookworms and whipworms, as well as other parasitic nematodes that are responsible for infections of nearly one-quarter of all humans (Cox, 2004; Bañuls et al., 2013). Yet, while Sydney Brenner promoted *C. elegans* as a model system to study neurobiology (Corsi et al., 2015), and many mammalian PDEs are associated with neurological conditions (Bender and Beavo, 2006; Maurice et al., 2014), surprisingly few studies have adequately examined the roles of these six PDEs in *C. elegans* or other nematode biology.

Some of the nematode research carried out using drugs that target mammalian PDEs suffers from one of two suppositions. First, there appears to be an assumption that nematode PDE gene numbers match to mammalian family numbers. This is true for *pde-1*, *pde-2, pde-3*, and *pde-4*, however *pde-5* encodes a member of the PDE10 family, while *pde-6* encodes a member of the PDE8 family. Thus, it is not clear how to interpret a study that features the impact of the PDE5 inhibitor Tadalafil on whipworms and hookworms, as these nematodes lack both PDE5 and PDE11 homologs (Tyagi et al., 2019), the only two mammalian PDE families for which Tadalafil displays any significant activity (Porst, 2002; Weeks et al., 2005). It is hard to imagine that such a highly selective drug as Tadalafil can inhibit a nematode PDE1, PDE2, PDE3, PDE4, PDE8, or PDE10 enzyme, and it seems as likely that it could be acting on a non-PDE target. Second, similar to the faulty logic that BRL50481 should be equally effective against both PDE7A and PDE7B (Smith et al., 2004), studies generally assume that a potent inhibitor of a mammalian PDE will be effective on a nematode homolog. This idea was clearly refuted in a study showing that the *C. elegans* PDE4 enzyme is 159-fold less sensitive to the PDE4 inhibitor Roflumilast and 77-fold less sensitive to the PDE3/4 dual specificity inhibitor Zardaverine, relative to mammalian PDE4 (Schuster et al., 2019). Thus, it seems largely unknown what effect inhibitors of individual nematode PDEs would have on these organisms.

To really determine if inhibiting a nematode PDE could produce a beneficial effect by reducing viability or fertility, one should examine the PDEs individually to identify a potent, cell-permeable inhibitor of each PDE and then examine the impact of the compound on the nematode. Potent inhibitors could be identified biochemically, however this would be an expensive endeavor that might not be seen as warranted by pharmaceutical companies or would require a substantial grant for academic labs. In addition, if the compounds fail to have an effect on *C. elegans*, it may actually be due to a lack of cell permeability, which would require even more research to develop cell-permeable derivatives of the original hit compounds. In contrast, the expense of cloning these genes into *S. pombe* expression vectors and building strains expressing them for the detection of inhibitors is quite modest, especially in this age of gene synthesis (Kunjapur et al., 2018). As we have already generated collections of compounds from HTSs against mammalian PDE4A and PDE4B, we intend to enter this arena by cloning the *C. elegans pde-4* gene and screening our panel of compounds for the ability to confer 5FOA^R^ growth. It is actually surprising how few studies of *C. elegans* PDEs have been carried out, given what a powerful multicellular model organism this is for genetic research. One study that caught our attention pointed out the relative under-representation of *pde-4* mutations in a mutant screen (one mutant observed when 40 were expected), a hallmark of essential genes (Charlie et al., 2006). Thus, if we can identify potent PDE4 inhibitors among our hit compounds, we can then determine their impact on *C. elegans* viability and fertility, without the concern about cell permeability that accompanies the use of compounds discovered by *in vitro* enzyme assays. Results from treating worms with these compounds could serve as justification for constructing strains expressing *pde-4* genes from parasitic nematodes for use in HTSs to identify more selective compounds that could be developed into drugs to treat infections by these organisms. Similarly, we could investigate the other five *C. elegans* PDEs by cloning and expressing them in *S. pombe*, identifying inhibitors, testing them on *C. elegans*, and then expanding into the homologs from parasites if warranted. While these other five PDEs may be less likely to serve as useful drug targets, the fiscal and technical barrier to testing these possible targets using this *S. pombe* expression platform is so low as to warrant consideration.

## Conclusion

The fission yeast platform described in this chapter has many favorable characteristics for the discovery and development of therapeutics that can target individual Class I PDEs or PDEs from more than a single family. Using this yeast-based 5FOA growth assay, one identifies compounds that target the PDE of interest, are cell permeable, and do not promiscuously bind proteins. Thus, while it is possible to overlook compounds with higher affinity for the target, these criteria select for compounds that already display several favorable druglike characteristics. Adding a new PDE to this platform is remarkably inexpensive, with the cost of cloning a 2 kb ORF at less than $200 using synthetic DNA and reagents already present in the laboratory. Meanwhile the HTS itself is also very affordable using 384-well clear plates with lids and a straightforward measurement of optical density to assess growth. However, as we have already demonstrated, these very affordable methods have allowed us to identify effective and selective PDE inhibitors that produce biologically relevant responses when used in mammalian cell culture, even before any medicinal chemistry has been carried out to develop more druglike compounds. These initial hit compounds have significant potential to lead to the development of therapeutics that target either mammalian PDEs to treat inflammatory disease or parasite PDEs to treat infections by parasitic nematodes, an area that has been poorly explored thus far.
